# How Microbes Shape Their Communities? A Microbial Community Model Based on Functional Genes

**DOI:** 10.1016/j.gpb.2018.09.003

**Published:** 2019-04-23

**Authors:** Xiaoqing Jiang, Xin Li, Longshu Yang, Chunhong Liu, Qi Wang, Weilai Chi, Huaiqiu Zhu

**Affiliations:** 1 State Key Laboratory for Turbulence and Complex Systems, Department of Biomedical Engineering, College of Engineering, Peking University, Beijing 100871, China; 2 Center for Quantitative Biology, Peking University, Beijing 100871, China; 3 Peking University-Tsinghua University-National Institute of Biological Sciences Joint Biological (PTN) PhD Program and College of Life Sciences, Peking University, Beijing 100871, China; 4 Center for Protein Science, Peking University, Beijing 100871, China

**Keywords:** Metagenomics, Dynamics model, Community structure, Acid mine drainage, Human gut microbiota

## Abstract

Exploring the mechanisms of maintaining microbial **community structure** is important to understand biofilm development or microbiota dysbiosis. In this paper, we propose a functional gene-based composition prediction (FCP) model to predict the population structure composition within a microbial community. The model predicts the community composition well in both a low-complexity community as **acid mine drainage** (AMD) microbiota, and a complex community as **human gut microbiota**. Furthermore, we define community structure shaping (CSS) genes as functional genes crucial for shaping the microbial community. We have identified CSS genes in AMD and human gut microbiota samples with FCP model and find that CSS genes change with the conditions. Compared to essential genes for microbes, CSS genes are significantly enriched in the genes involved in mobile genetic elements, cell motility, and defense mechanisms, indicating that the functions of CSS genes are focused on communication and strategies in response to the environment factors. We further find that it is the minority, rather than the majority, which contributes to maintaining community structure. Compared to health control samples, we find that some functional genes associated with metabolism of amino acids, nucleotides, and lipopolysaccharide are more likely to be CSS genes in the disease group. CSS genes may help us to understand critical cellular processes and be useful in seeking addable gene circuitries to maintain artificial self-sustainable communities. Our study suggests that functional genes are important to the assembly of microbial communities.

## Introduction

There has never been a better time to investigate microbial communities [1]. Not only is the influence of microbial communities on biogeochemical cycles, Earth’s climate, and human health beginning to be understood, but also cultivation-independent omics techniques as well as high-throughput sequencing technologies are driving a rapid revolution of our knowledge on the diversity and complexity of microbial communities in natural environments [2]. Microorganisms are probably the most diverse organisms and microbial community structures are very important to understand ecosystem functions [3]. However, many issues remain elusive, such as the mechanisms underlying microbiota development and maintenance [4]. Maintaining the structure of microbial communities is critical to ecosystem and human health. On the one hand, there are great differences in the microbial community structure between lowly and highly metal contaminated samples [5]. On the other hand, gut microbial dysbiosis is associated with various diseases, including irritable bowel syndrome (IBS) [6], [7], [8] and depression [9]. Accordingly, understanding the development and maintenance of microbiota may be helpful in providing feasible strategies for bioremediation and disease therapy.

Many studies on microbial communities were focused on the influence of various environmental factors on the microbial community assemblage, such as the imposed treatments [10], biochar [11], substrate inputs [12], and pH [13]. However, the roles of functional genes in community structure remain unknown. Functional genes are important to confer the metabolic phenotypes of microbes, leading to complex ecological interaction, which is a major determinant of microbial community structure [14]. Admittedly, it has long been known about so-called essential genes for microbes, *i.e.*, the genes of an organism or of a genome that are widely considered to be crucial for its survival under given conditions [15]. Current studies on essential genes have made great progress and improved our knowledge of their associated biological functions [16], [17], [18], [19]. However, in natural environments, more than one type of microorganism lives together within a community, interacting with each other and exhibiting various social behaviors. In practice, the essential genes have not yet provided us an insight into the way to shape a microbial community for many a microorganism in natural environments. Thus, functional genes crucial for shaping community structure (we proposed as the community structure shaping genes, *i.e.*, CSS genes), rather than the essential genes, are more expected to reveal the impacts of genes on development and maintenance of community structure in natural environments.

A well-known limitation of current studies on the microbial community structure is that monitoring the dynamics of community structure over time, even with an appropriate experimental design, is still difficult and cost-consuming [20]. Fortunately, mathematical models offer an access to study the microbial communities that are difficult to be cultivated in the lab. Several methods are available for modeling the dynamics of microbiota. The microbial assemblage prediction (MAP) [21], a predictive model based on artificial neural networks, has achieved much. However, this model takes biological processes as black boxes, taking less account of the inner workings or parts. Rigorous mathematical models are more conducive to realizing the fundamental elements of microbial populations. The generalized Lotka–Volterra model [22], [23] and generalized additive model [24] are commonly used and have made much progress. However, they fail to show good prediction and have certain known limitations [25], [26]. The generalized Lotka–Volterra equations do not capture mutualisms and some other types of relationships [26], whereas the generalized additive model assumes that the relationships are additive, which may not be realistic for complex ecosystems [26]. The replicator dynamics model [27] is the first and most successful model to study classic evolutionary game theory and has been used extensively in many fields, such as population genetics, biochemical evolution, and sociobiology. However, these dynamic models do not take environmental factors into consideration and assume constant population size, which may not hold for microbial populations [28]. In summary, these methods have shed light on modeling microbial communities, while their limitations deserve a serious concern in state-of-the-art methods, such as poor performance and doubtful assumptions.

In this paper, we proposed a modified replicator dynamics model, functional gene-based composition prediction (FCP) model, to predict the population structure composition within a microbial community. Compared to the classical replicator dynamics models, FCP has made three main improvements by (1) explicitly analyzing the dynamics of microbes with variable population size; (2) linking environmental parameters, microbial community structure, and functional characteristics; and (3) using the dissimilarity of taxonomic units at the functional level based on gene annotation of metagenomic sequences and environmental variations to quantify the fitness. Fitness is the most central parameter in replicator dynamics models and its quantification has been a long-time goal for evolutionary game theory [29]. The fitness describes the viability of microbes as compared to that of other microorganisms in the community. The interspecific competition and environmental variations have promoted the evolution of microbial community, but in opposite directions [30]. Environmental filtering increases functional similarity within communities while competition for limited resources tends to decrease functional similarity [30]. Consequently, unlike classical replicator dynamics models that often merely consider microbial interactions, we used both microbial interactions and environmental variations to quantify fitness. In summary, we set out to design and test a model focused on predicting microbial community assemblages. Furthermore, we defined functional genes that are indispensable for shaping microbial community structure as the CSS genes. With the application of the FCP model, we identified CSS genes and investigated which parts of functional genes were critical for shaping the community structure. CSS genes may be useful in seeking addable gene circuitries to maintain artificial self-sustainable communities and treating diseases related to microbiota dysbiosis. Our model provides a viewpoint of the relationships between functional genes and microbial community structure, and our study suggests that functional genes are key to the assembly of microbial communities.

## Results

### The overview of AMD microbial communities

**Figure 1:**
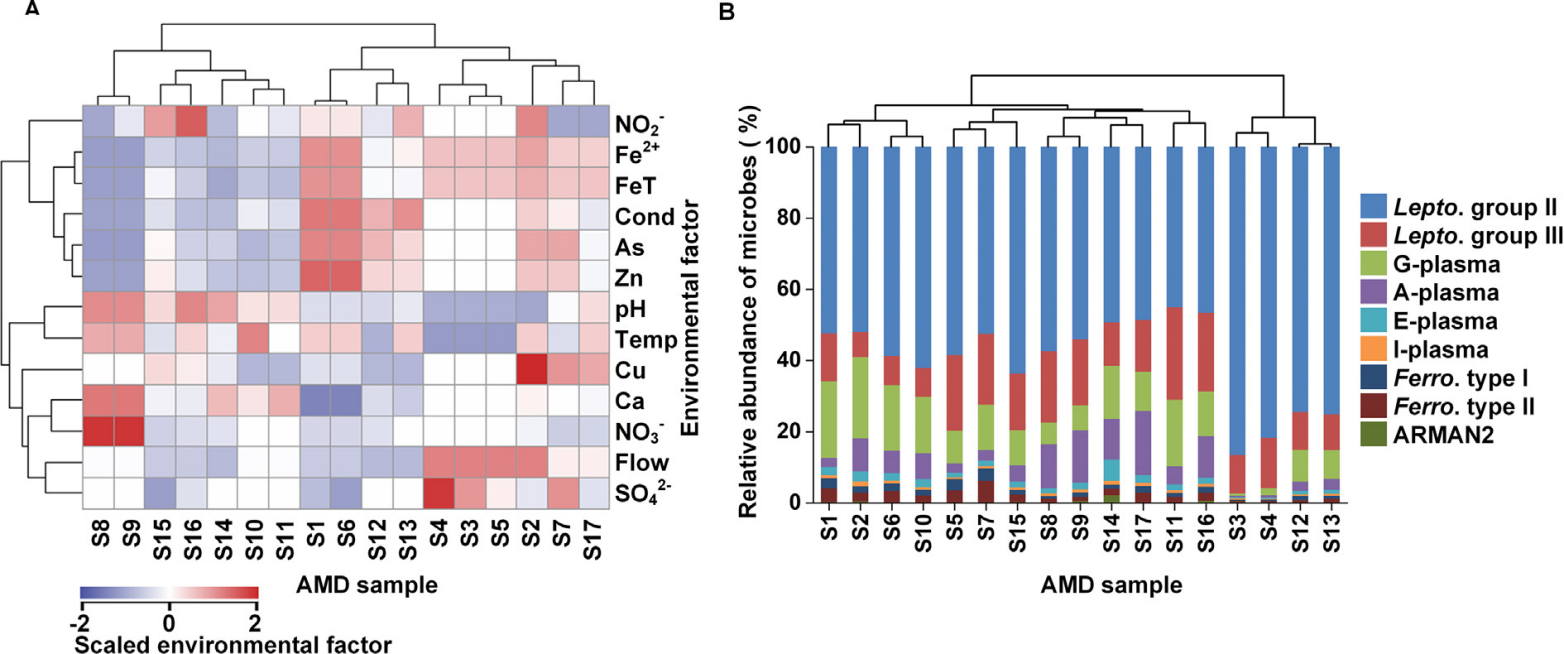
**Hierarchical cluster analysis of AMD samples** **A**. Hierarchical cluster analysis of environmental factors of AMD samples. Environmental factors including solution discharge rate (Flow, l/min), pH, temperature (Temp, °C), electrical conductivity (Cond, mS/cm), and the concentrations of ferrous (Fe^2+^, M)/ferric and ferrous (FeT, M)/ copper (Cu, mM)/arsenic (As, mM)/zinc (Zn, mM)/calcium (Ca, mM)/ sulfate (SO_4_^2−^, M)/nitrate (NO_3_^−^, nM)/nitrite (NO_2_^−^, nM) were standardized before hierarchical clustered. The standardized values of environmental factors are color-coded in the heatmap, with larger values in red and smaller values in blue. **B.** Hierarchical cluster analysis of microbial community composition of AMD samples. A-plasma, E-plasma, G-plasma, and I-plasma represent Thermoplasmatales archaeon A-plasma, E-plasma, G-plasma and I-plasma, respectively; *Lepto.* group II, *Lepto.* group III, *Ferro*. type I, and *Ferro*. type II indicate *Leptospirillum* group II, *Leptospirillum* group III, *Ferroplasma* type I, and *Ferroplasma* type II, respectively. ARMAN2 are from the archaeal Richmond Mine acidophilic nanoorganisms (ARMAN) lineages. The results show that 17 samples could be classified into two groups. The group (S3/S4/S12/S13) with higher percentage of *Leptospirillum* group II is in the early developmental stage and the other group is in the late succession stage. AMD, acid mine drainage.

Since predicting microbial community assemblages is often limited by the inherent complexity of biological systems, we performed the current study by analyzing acid mine drainage (AMD) metagenomic sequences as the model metagenome data. AMD biofilm is a relatively self-contained and low-complexity system [31]. The genomes of AMD microorganisms were sequenced with high-throughput sequencing strategies [32]. After data preprocessing (see methods), totally 17 AMD samples, characterized by acidity, heat, and high concentrations of heavy metals, had been collected from the air-solution interface by Banfield and colleagues [32]. A broad variety of environmental factors at each sample site had been measured [32] and clustered. As shown in Figure 1A, temperature and pH were clustered in one group, revealing a close correlation between these two factors. Proteins from the chemoautotrophic iron-oxidizing bacteria *Leptospirillum* group II (59.48 ± 12.54%) were predominantly present in almost all samples (Figure 1B). The 17 samples have been clustered into two groups, representing different developmental stages (Figure 1B). The classification results are quite similar to those reported previously [32]. The group with a high relative abundance of *Leptospirillum* group II (79.41 ± 5.70%) was in the early developmental stage. The other group was in mature stage and had lower relative abundance of *Leptospirillum* group II (53.35 ± 5.34%, Student’s *t* test, *P* < 10E−9).

**Figure 2:**
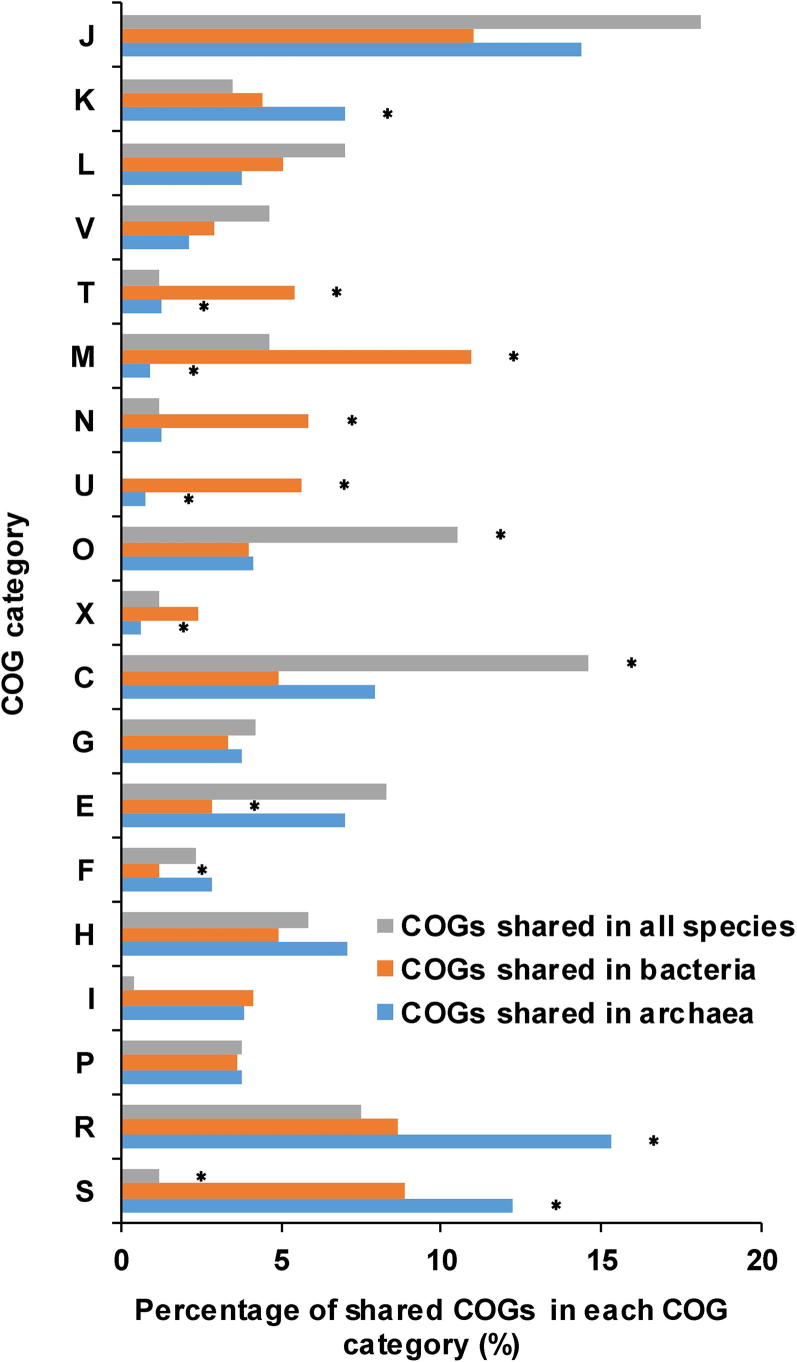
**Comparison of COG distributions in AMD samples** Comparisons of the distributions of COGs shared in all species, bacteria, and archaea, respectively. The vertical axis shows the different COG categories and the percentage of shared COGs in each category is shown on the horizontal axis. Asterisks indicate that the enrichments are significant (Fisher’s exact test, *P* < 0.05). COG refers to Clusters of Orthologous Groups. The COG categories are listed as follows. J, translation, ribosomal structure and biogenesis; K, transcription; L, replication, recombination and repair; V, defense mechanisms; T, signal transduction mechanisms; M, cell wall/membrane/envelope biogenesis; N, cell motility; U, intracellular trafficking, secretion, and vesicular transport; O, posttranslational modification, protein turnover, chaperones; X, mobilome: prophages, transposons; C, energy production and conversion; G, carbohydrate transport and metabolism; E, amino acid transport and metabolism; F, nucleotide transport and metabolism; H, coenzyme transport and metabolism; I, lipid transport and metabolism; P, inorganic ion transport and metabolism; R, general function prediction only; S, function unknown.

To examine the gene distribution in AMD community, we aligned the near-complete genomes of nine species in AMD to all predicted peptides in the Clusters of Orthologous Groups (COG) protein database [33] (http://www.ncbi.nlm.nih.gov/COG/). By blasting to totally 4631 COGs in the database, we found that AMD samples had 7380 different genes which were classified into 1998 COGs. The gene function annotation indicated the difference between bacterial and archaeal genomes (Figure 2). About 4.31% of COGs were shared in all microbes and enriched in the COG categories of J (translation, ribosomal structure and biogenesis), C (energy production and conversion), and O (post-translational modification, protein turnover and chaperones), reflecting the similarities in translation and post-translational modification of bacteria and archaea. Genes in the categories M (cell wall/membrane/envelope biogenesis), N (cell motility), U (intracellular trafficking, secretion and vesicular transport), and T (signal transduction mechanisms) were remarkably shared in bacteria (Fisher’s exact test, *P* < 0.05). Most of these genes were involved in communication and motility, allowing bacteria to respond to environmental changes timely. Metabolism-related genes were rarely shared in bacterial genomes. However, there came to almost the opposite conclusions for archaea, whose genomes mostly shared metabolism but lacked the COG categories T (signal transduction mechanisms), M (cell wall/membrane/envelope biogenesis), and U (intracellular trafficking, secretion and vesicular transport). In summary, bacterial genomes shared more genes related to responses to extreme acidic environments while archaeal genomes shared more genes involved in metabolism.

### Relationships between microorganisms and environmental factors in AMD samples

**Figure 3:**
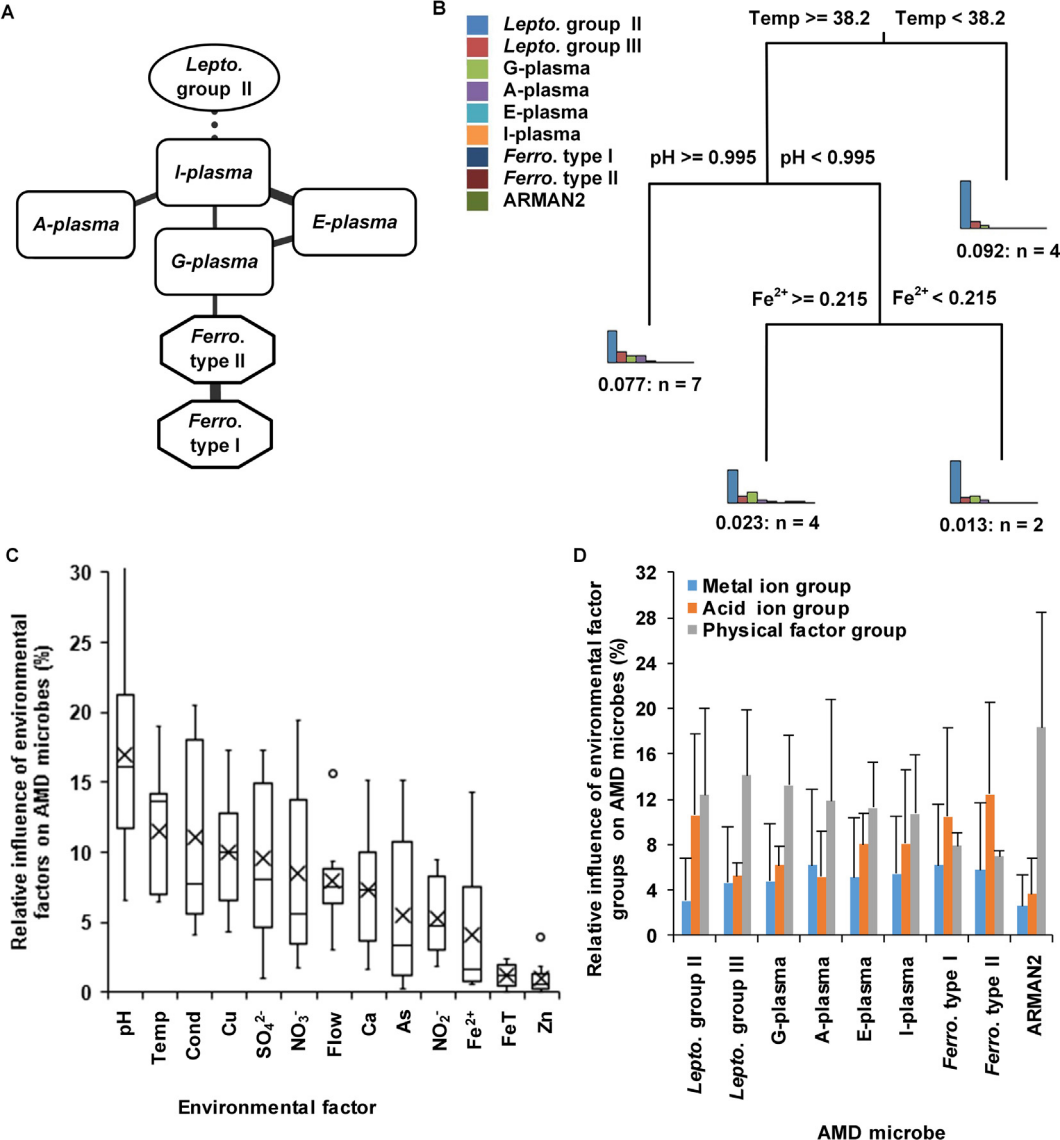
**Relationships between relative abundances of microorganisms and environmental factors in AMD samples** **A.** Social relationship network in AMD biosystem. Only statistically significant edges (*P* < 0.05, after Bonferroni correction, correlation coefficient ≥0.65) were retained. Dotted lines reflect negative relationship between different microbes and solid lines represent positive ones. The thicker lines indicate higher correlation coefficients, *i.e.*, stronger relationships between microorganisms. **B.** Relationships between community structure and environmental factors in AMD samples with MRT model. **C.** Relative influence of environmental factors on microorganisms using GBM method. The circles represent the outlier values and black crosses show the mean influence of the corresponding environmental factors on microorganisms. **D.** Relative influence of environmental factor groups on microorganisms. Environmental factors are divided into three groups: physical factor group (pH, temperature, flow, and conductivity), acid ion group (SO_4_^2−^, NO_3_^–^, and NO_2_^–^), and metal ion group (Fe^2+^, FeT, Zn^2+^, Cu^2+^, As^3+^, and Ca^2+^). Relative effects of each group on microorganisms calculated using GBM models are presented. MRT, multivariate regression tree; GBM, gradient boosting machine.

We investigated the relationships among the relative abundances of microorganisms in AMD samples with the compositionality corrected by renormalization and permutation (CCREPE) algorithm (http://huttenhower.sph.harvard.edu/ccrepe). Statistically significant edges (*P* < 0.05, after Bonferroni correction, correlation coefficient ≥0.65) are shown (Figure 3A). G-plasma, E-plasma, I-plasma, and A-plasma, members of the order Thermoplasmatales, were clustered in one group. The relative abundance of G-plasma was closely related to that of *Ferroplasma* type II. A positive correlation between the relative abundances of *Ferroplasma* type II and *Ferroplasma* type I was also found. These observations suggest a potential positive correlation within genomes of allied species. However, the relative abundance of the dominant species, *Leptospirillum* group II, had a negative correlation with that of I-plasma, thus exhibiting a potential negative correlation with most of the remaining microorganisms. Since that *Leptospirillum* group III and the archaeal Richmond Mine acidophilic nanoorganism (ARMAN) lineage 2 (ARMAN2) were not present in this network, their relative abundances showed poor associations with those of other microorganisms in AMD samples. The common positive correlations among closely-related species and negative relationships in distantly-related species were achieved in part by environmental filtering, which tended to cluster similar functions and disperse dissimilar functions. The 16S rRNA sequences and whole genome annotation results between *Leptospirillum* group II and III had a strong correlation, and the same existed between A-plasma and G-plasma and between E-plasma and G-plasma. However, there were no significant direct relationships in their relative abundances. In addition, the coefficient of variation (the ratio of standard deviation to average) of *Leptospirillum* group III and ARMAN2 were 0.38 and 1.36, respectively, which is much greater than that of *Leptospirillum* group II (as 0.21). This indicates that the relative abundances of *Leptospirillum* group III and ARMAN2 are not constant. The two points above suggest that the community composition in AMD samples is not only affected by environments but also influenced by other factors, for example, interspecific competition.

To measure the relative influence of different environmental factors on microbial structure in AMD samples, we conducted the multivariate regression tree (MRT) [34] analysis (Figure 3B). Herein temperature appeared to be a strong predictor of community structure, because samples with high temperature were distinguished from those with moderate temperature. *Leptospirillum* group II had a relatively low abundance (54.98 ± 8.07%) in extremely hot environments (temperature ≥38.2 °C) but was absolutely dominant (74.11 ± 14.34%) under moderate temperature conditions (temperature <38.2 °C). This indicates that as AMD biofilms mature, they become increasingly heated. The energy might come from series of complex chemical reactions in AMD biofilms. Furthermore, we used the gradient boosting machine (GBM) method [35] to measure the different contributions of environmental factors to the relative abundance of each microorganism. Our results demonstrated that pH and temperature are the two most influential variables (Figure 3C). The low relative impact of Zn^2+^, Fe^2+^, and FeT concentrations on all microorganisms showed their limited contributions to the dissimilarity of community structure. We classified the environmental factors into three groups: physical factor group (pH, temperature, flow, and conductivity), acid ion group (SO_4_^2−^, NO_3_^−^, and NO_2_^−^), and metal ion group (Fe^2+^, FeT, Zn^2+^, Cu^2+^, As^3+^, and Ca^2+^). The results showed that physical factor group had higher impact on these microorganisms than acid ion group (Figure 3D) (Student’s *t* test, *P* < 0.05), while metal ion group had the lowest impact (Student’s *t* test, *P* < 0.007). Previous studies [36] illustrated that pH was the major factor contributing to community difference in Southeast China AMD samples and Fe^2+^ and Fe^3+^ were also relative important factors. Herein we found that pH and temperature were closely related (Figure 1A) and both were major factors. However, different from previous studies [36], our results showed that Fe^2+^ and FeT had little influence on most species.

### Prediction of microbial community composition in both AMD and human gut microbiota samples

**Figure 4:**
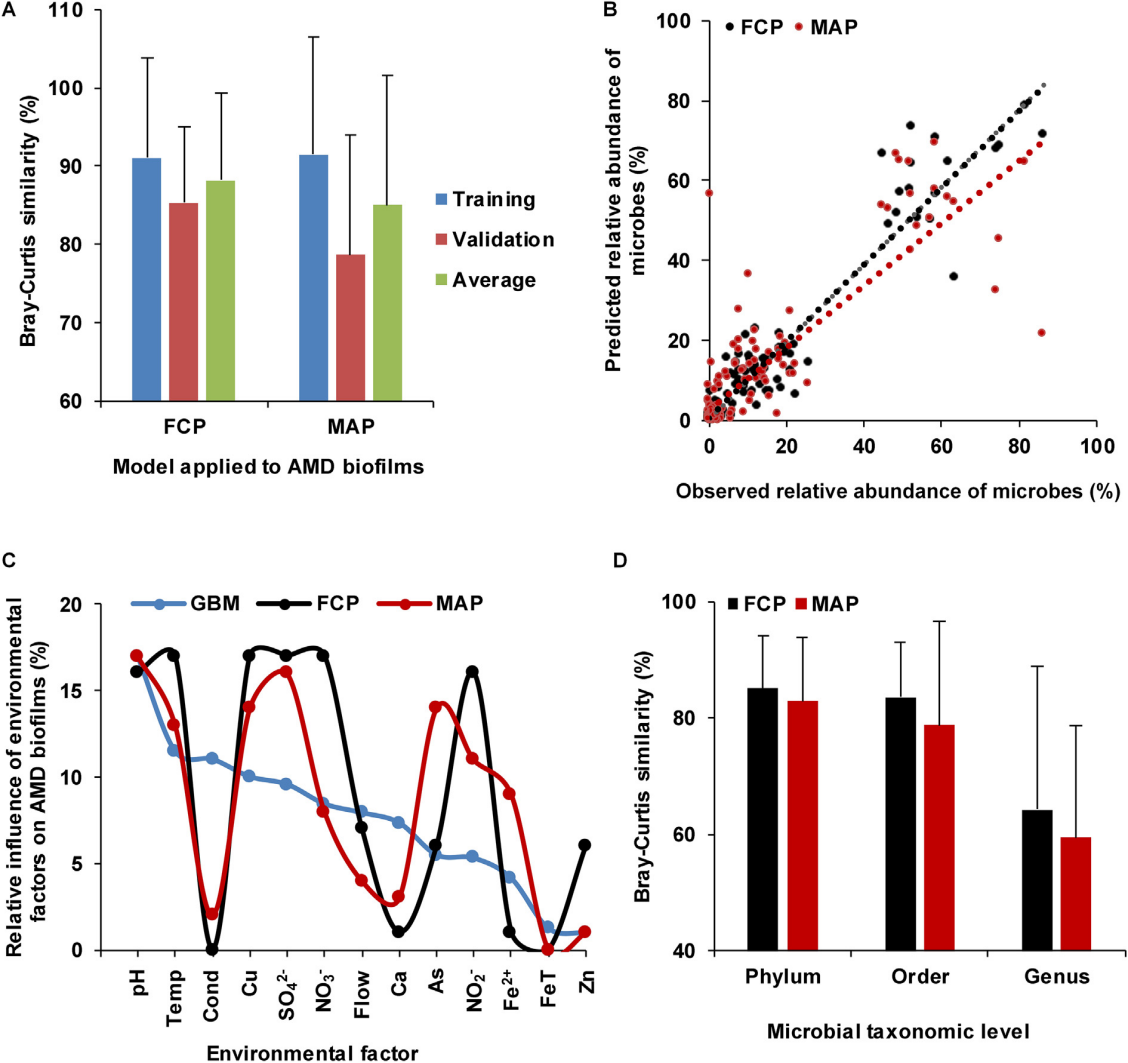
**Comparison of prediction accuracies between the FCP and MAP methods** **A.** Prediction accuracies of the FCP and MAP methods in AMD samples. The prediction accuracies for the training dataset and validating dataset in AMD samples are measured using Bray–Curtis similarity, with the average accuracies also shown. **B.** Cross-validation of the predicted relative microbial abundances with MAP and FCP methods in AMD communities. The linear regression of the FCP model is expressed as *y* = 0.96*x* + 0.003 (*R*^2^ = 0.92) and that of the MAP model is expressed as *y* = 0.79*x* + 0.03 (*R*^2^ = 0.72), respectively. **C.** Relative influence of environmental factors on AMD biofilms using GBM, FCP, and MAP methods. **D.** Comparison of prediction accuracies between the FCP and MAP models in human gut microbiota samples. The accuracies are measured using Bray–Curtis similarity. FCP, functional gene-based composition prediction; MAP, microbial assemblage prediction.

We then used FCP to simulate how community composition responds to environmental factors. The environmental factors cause allied species to cluster, whereas interspecific competition makes them disperse, thus forming dynamic balance in microbial communities. Using both interspecific interaction and environmental information to quantify the driving force of community development, FCP model achieved a satisfactory prediction (Figure 4A) in AMD samples. The MAP model, which has proven to be effective in prediction of microbial assemblages [21], was applied to AMD samples as well. The cross-validation of predicted values showed that the FCP model (*R*^2^ = 0.92, equation of linear regression: *y* = 0.96*x* + 0.003, Bray–Curtis similarity = 85.32 ± 9.68%) performed better than (one-tailed Student’s *t* test, *P* = 0.032) the MAP model (*R*^2^ = 0.72, *y* = 0.79*x* + 0.03, Bray–Curtis similarity = 78.65 ± 15.30%) (Figure 4B). Therefore, our FCP model demonstrated a higher degree of accuracy and smaller variance than the MAP model. The relative influence of environmental factors on AMD biofilms predicted using the FCP, MAP, and GBM methods is shown in Figure 4C (correlation coefficient_(FCP, MAP)_ = 0.75, *P* = 0.0034; correlation coefficient_(FCP, GBM)_ = 0.52, *P* = 0.069; correlation coefficient_(MAP, GBM)_ = 0.59, *P* = 0.035). The high correlation coefficient of the relative impact of environmental variables showed good consistency between the MAP and FCP methods.

To illustrate the effectiveness and applicability of the FCP model, we further applied it to human gut microbiota (Figure 4D) from healthy and diseased individuals. IBS is one of the most prevalent functional gastrointestinal disorders, influencing 5%–11% of the population in most countries [37]. The comorbidity of IBS with depression is common [38]. Alterations in the gut microbiota have been found relevant to both IBS and depression [39]. Thus, it is important to understand how gut microbiome changes in persons with IBS and depression. We have collected fecal samples from 54 individuals [38], including 21 patients with IBS, 6 with depression, 12 with comorbid IBS and depression, and 15 health controls (Table S1). In addition, 14 variables were measured, including height, weight, pain threshold, and concentrations of relevant molecules (Table S2). These samples were divided into two sets, one for model training and another for validation. For effective validation, each set included samples from the IBS, depression, comorbidity, and health control groups. The prediction using our FCP model (phylum: Bray–Curtis similarity = 85.08 ± 9.02%, *R*^2^ = 0.72; order: Bray–Curtis similarity = 83.55 ± 9.53%, *R*^2^ = 0.83; and genus: Bray–Curtis similarity = 64.16 ± 24.58%, *R*^2^ = 0.40) appeared to be better than (one-tailed Student’s *t* test, phylum: *P* = 0.15; order: *P* = 0.06; and genus: *P* = 0.10) that using the MAP model (phylum: Bray–Curtis similarity = 82.88 ± 10.91%, *R*^2^ = 0.70; order: Bray–Curtis similarity = 78.76 ± 17.92%, *R*^2^ = 0.74; and genus: Bray–Curtis similarity = 59.41 ± 19.11%, *R*^2^ = 0.28) at the phylum, order, and genus levels, respectively.

Consequently, the FCP model developed based on functional gene usage distribution was validated for both low-complexity and complicated microbial communities. The performance of FCP model was better than MAP model in both two datasets. In addition, the MAP model might generate a few isolated nodes and thus was unable to predict corresponding microorganisms well. Meanwhile, abnormal results were observed in some samples when predicting using the MAP mode and these outliers had to be removed (nine outliers at the level of order and six at the genus level in 54 human gut microbiota samples), while there were no such cases when using our FCP model. Different from the MAP model that takes a black-box view, our FCP model has informative formulas and thus has the potential of grasping the intrinsic mechanisms of complex microbial communities.

### Identification of CSS genes crucial for shaping community structure in AMD and human gut microbiota samples

**Figure 5:**
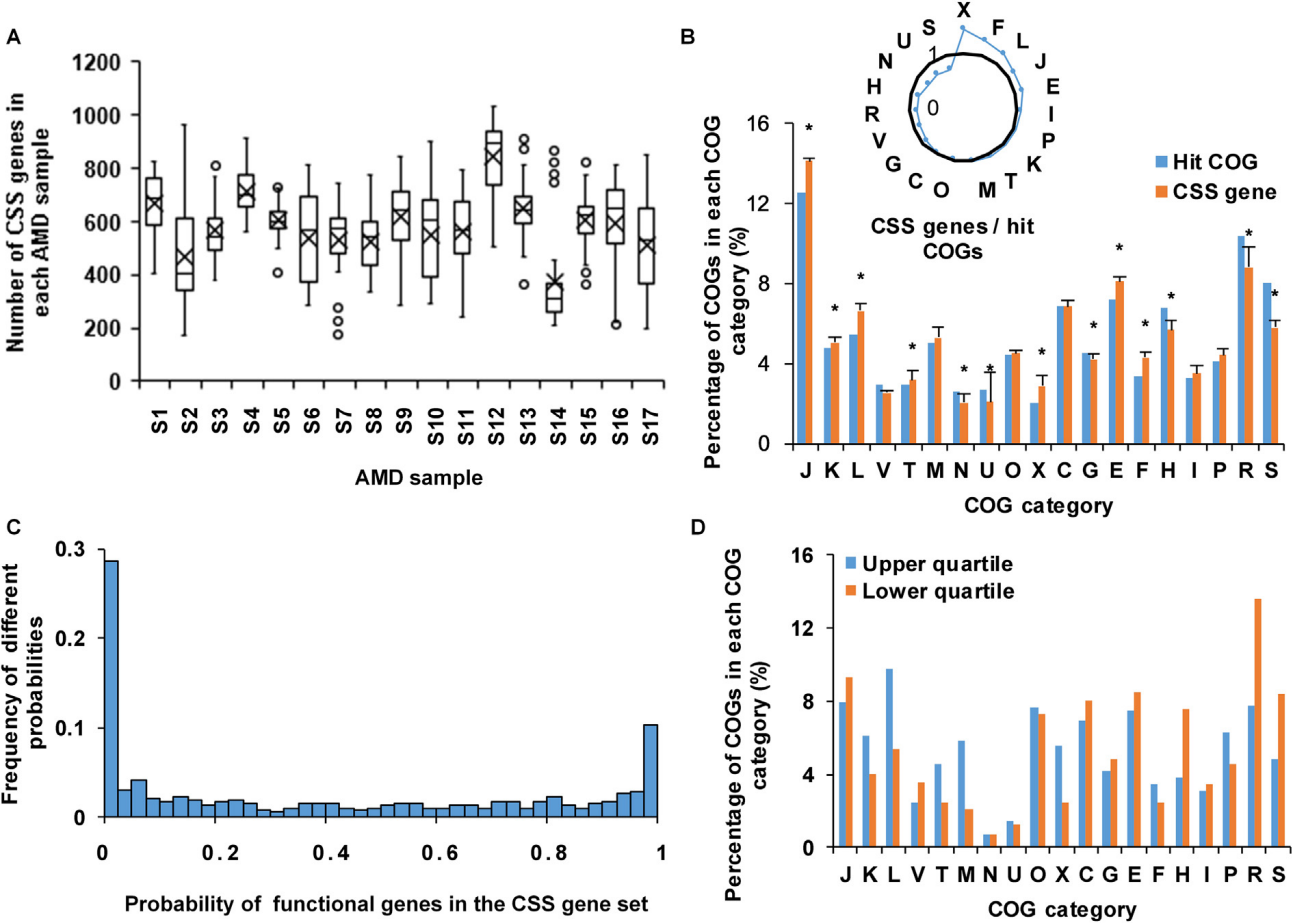
**Analyses of CSS genes in AMD samples** **A.** Numbers of CSS genes in AMD samples. The circles represent the outlier values and black crosses show the average numbers of CSS genes in corresponding samples. **B.** Comparison of the distribution of CSS genes with 1998 hit COGs. The radar map shows the relative size of CSS genes and hit COGs in each COG category. The asterisks show that the enrichments are significant (Fisher’s exact test, *P* < 0.05). **C.** The distribution of probabilities of functional genes in the CSS gene set. **D.** The distribution of functional genes whose probabilities in the CSS gene set are in the first 25 percentage (upper quartile) and the last 25 percentage (lower quartile). Details of the COG categories are provided in the legend of Figure 2. CSS, community structure shaping.

With all annotated protein coding genes, the FCP model constructs the microbial community based on the functional gene usage. A further question of great interest is which part of these genes is important to shape such a microbial community. Clearly this part of genes should be distinct from the set of essential genes. To test this, we defined this part of genes as CSS genes in this study. Using the FCP model and metagenomic data, we developed a selection method to identify CSS genes (see Methods). Considering that many genes have the same or similar functions, we measured CSS genes in the unit of homologous genes according to the COG database. Applying the selection method to AMD samples (Table S3, Figure 5), we identified 583.3 ± 103.3 CSS genes (Figure 5A). Among the samples, sample S14 had the lowest number of CSS genes, amounting to 375 CSS genes, while sample S12 had the highest number of CSS genes, amounting to 841 CSS genes.

As mentioned above, we finally identified 1998 COGs after alignments in AMD samples. Now we compared the 1998 COGs with CSS genes to discover enriched or depleted functions in the CSS genes. The remarkable enrichment of CSS genes in the COG category X (mobilome: prophages, transposons) revealed that gene exchange and recombination were important in AMD samples (Figure 5B). Previous studies [31] illustrated that AMD communities might have a high mutation rate or gene conversion frequency. One of the interesting findings is that 8/20 transposases had a high probability (>0.975) to be CSS genes. Transposases, regarding as selfness genes, might mobilize or activate genes that induce advantageous rearrangements [40] and enhance their hosts’ fitness [41]. Therefore they are important to community structure. Meanwhile, these 1998 COGs had 846 different profiles of hit numbers for nine species, and the probability distribution of each profile in the CSS gene set in 17 AMD samples to a U shape (Figure 5C). The upper and lower quartiles of this U-shape distribution were 0.01 and 0.79, respectively, indicating that a large percentage of genes are always CSS genes and some genes are always not. Compared to genes with low probabilities in the CSS gene set, the genes with high probabilities were involved in the categories M (cell wall/membrane/envelope biogenesis), X (mobilome: prophages, transposons), T (signal transduction mechanisms), and L (replication, recombination and repair) (Figure 5D). These data indicate that genes related to exchange and communications are important to shape community structure in all 17 samples.

**Figure 6:**
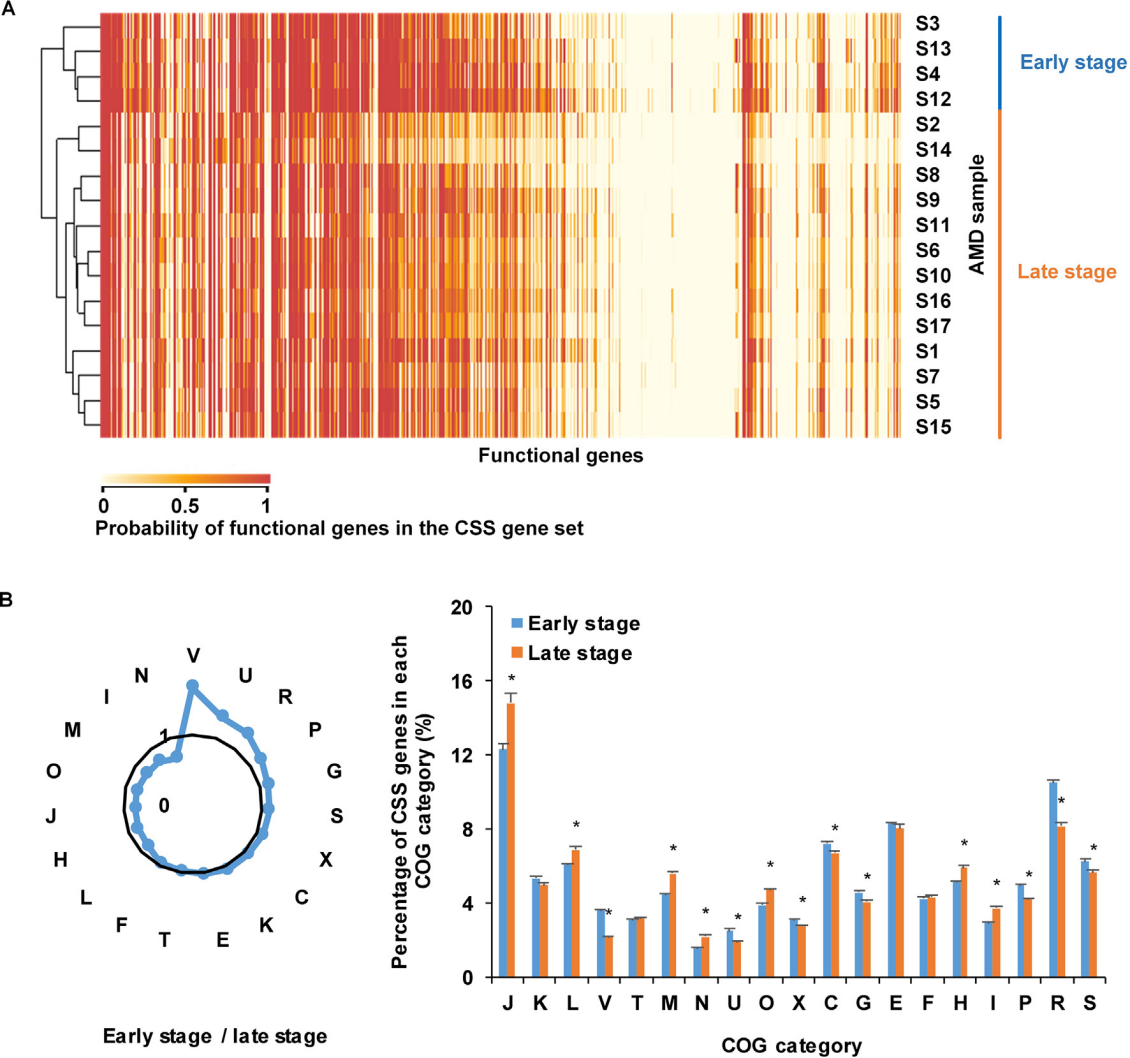
**Comparison of CSS genes in AMD samples at the early and late succession stages** **A.** Cluster analysis of probabilities of functional genes in the CSS gene set in AMD samples. The color in the heatmap shows the probabilities of functional genes in the CSS gene set, with larger values in red while smaller values in yellow. The results show that these functional genes are clustered into two groups. **B.** Comparison of the relative magnitudes of CSS genes in the early and late stage samples. The radar map shows the relative size of CSS genes in the early and late succession stage samples in each COG category. The asterisks show that the enrichments are significant (Fisher’s exact test, *P* < 0.05). Details of the COG categories are provided in the legend of Figure 2.

Our analysis further showed that the number of CSS genes increased with the relative abundance of bacteria (correlation coefficient = 0.60, *P* = 0.01) in AMD samples. The average number of CSS genes in the late succession stage samples (with 549.38 ± 74.74 COGs) was much smaller than that of early succession stage ones (with 693.42 ± 115.80 COGs). Furthermore, CSS genes in the early and late developmental stages were completely clustered into two groups (Figure 6A). It reveals that CSS genes were distinctly different at these two stages, possibly due to community physiological changes during ecological succession. Compared to the biofilms in the late succession stage, CSS genes involved in the COG categories V (defense mechanisms), U (intracellular trafficking, secretion, and vesicular transport), R (unknown functions), and P (inorganic ion transport and metabolism) were enriched in the early developmental stage biofilms (Figure 6B). In the late developmental stage samples, we found more CSS genes involved in the categories N (cell motility), I (lipid transport and metabolism), M (cell wall/membrane/envelope biogenesis), O (post-translational modification, protein turnover, and chaperones), and J (translation, ribosomal structure and biogenesis). These results substantially agree with previous studies [32], stating that proteins associated with physical and chemical stress defense, transcription, mobile genetic elements, and unknown functions were significantly overexpressed at the early stage, while proteins involved in motility, environmental signaling, chaperones and protein turnover, membrane biosynthesis, translation, and core metabolism were concentrated in mature biofilms.

**Figure 7:**
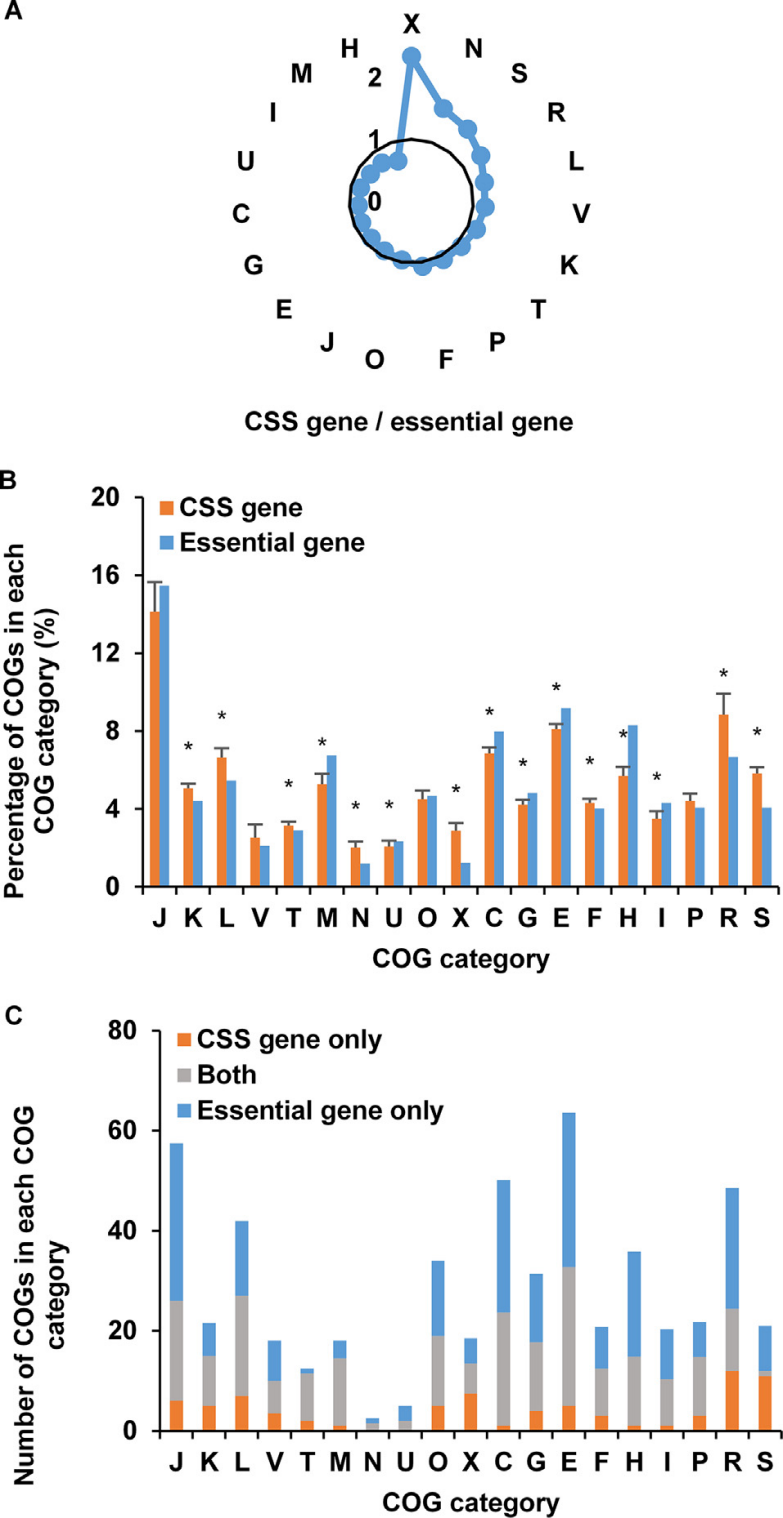
**Comparison of CSS genes and essential genes in AMD samples** **A.** Comparison of the relative size of CSS genes and essential genes in each COG category. **B.** Comparison of the distribution of CSS genes with essential genes in all COG categories. The small error bar illustrates the consistencies in all AMD samples. The asterisks show that the enrichments are significant (Fisher’s exact test, *P* < 0.05). **C.** The differences between CSS genes and essential genes in all COG categories. COGs that exist in both the CSS gene set and essential gene set are shown in gray. Blue and orange bars indicate genes that are specific to CSS gene set and essential gene set, respectively. Details of the COG categories are provided in the legend of Figure 2.

As mentioned above, we do not regard the CSS genes as the essential genes. To discover the differences among them, we compared CSS genes with the database of essential genes (DEG, http://www.essentialgene.org/) [15]. We found that CSS genes were involved in more gene functions about information communication, such as categories X (mobilome: prophages, transposons), N (cell motility), L (replication, recombination and repair), and V (defense mechanisms) (Figure 7A and B) than essential genes. More genes related to categories R (general function prediction only) and S (function unknown) were enriched in the CSS genes than essential genes. Out of the 1998 hit COGs, 672 COGs had unique hit number profile. Among them, 308 COGs had high probabilities (>0.5) in the CSS gene set and 472 COGs were found in DEG. There were 229 COGs shared by CSS gene set and DEG, and the permutation test showed that this overlap was significant (permutation time = 10,000, *P* = 0.01) (Table S4). The distribution of these 229 COGs revealed that some genes involved in metabolism and central dogma were both essential genes and CSS genes (Figure 7C). 79 COGs, which were probable CSS genes and not find in DEG, were enriched in the categories X (mobilome: prophages, transposons), L (replication, recombination and repair), and J (translation, ribosomal structure and biogenesis), which were related to central dogma and mobile genetic elements. 243 COGs, found in DEG but not in the CSS gene set, were mostly related to the categories J (translation, ribosomal structure and biogenesis), E (amino acid transport and metabolism), C (energy production and conversion), and H (coenzyme transport and metabolism). Among 672 COGs with unique hit number profile, there were 144 COGs with probabilities >0.5 in the CSS gene set in all 17 samples. These 144 COGs formed core CSS gene set, which were enriched in E (amino acid transport and metabolism), J (translation, ribosomal structure and biogenesis), and H (coenzyme transport and metabolism), with 30 COGs in E, 27 in J, and 17 in H categories, respectively. 74.31% (107/144) core CSS genes were found in DEG. The COGs that belonged to core CSS genes but not in DEG (totally 37 COGs) were mostly enriched in the categories S (function unknown), X (mobilome: prophages, transposons), and R (general function prediction only), with 7 COGs in S, 6 in X, and 5 in R categories, respectively.

We find that there are great differences in the contribution levels of each genome to CSS genes, essential genes, and all hit COGs. Among the total 1998 hit COGs, about 34.3% (680/1998) COGs were only present in bacterial genomes and 32.68% COGs (653/1998) in archaeal genomes in AMD community, indicating that the contribution levels of bacterial and archaeal genomes to all hit COGs were approximately equal. Bacterial genomes contributed much less to CSS genes than to all hit COGs, whereas archaeal genomes contributed more to CSS genes than to all hit COGs and to essential genes. Among 229 COGs shared in CSS gene set and DEG, 10.04% (23/229) COGs were included only in bacterial genomes and 29.26% (67/229) only appeared in archaeal genomes. Among 79 COGs which were only present in the CSS gene set, only 1.27% (1/79) COG was from bacterial genomes while 49.37% (39/79) COGs were only present in archaeal genomes. For the 243 COGs which were only present in DEG, 0.4% (1/243) COG was only in bacterial genomes, while 19.75% (48/243) were only in archaeal genomes. Therefore, despite of the low relative abundances of archaea, they contributed greatly to maintaining the community structure.

In the extreme acidic, heated, and high concentration of heavy metals content environment, resisting the pressure from the surroundings becomes one of the greatest challenges to microbes. The size of CSS gene set was decreased as biofilm matured and CSS genes involved in lipid transport and metabolism, cell motility, and membrane biogenesis were more abundant at the late developmental stage, indicating an increase in communication and motility in mature microbial communities. Compared to the essential genes, CSS genes were focused on genes exchanges and responses to extreme environments, as indicated by the discovery that CSS genes were significantly enriched in mobilome and defense mechanism. Meanwhile, CSS genes shared 229 COGs with essential genes and these COGs mainly were focused on metabolism and central dogma. These indicate that some metabolism-related genes were crucial for microorganisms no matter they were cultivated alone or inhabited in the natural environments with other microorganisms. Our study shows that CSS genes could reflect the selection pressure from environments and relationships between species. It also helps to understand important cellular processes that sustain life in the natural settings.

**Figure 8:**
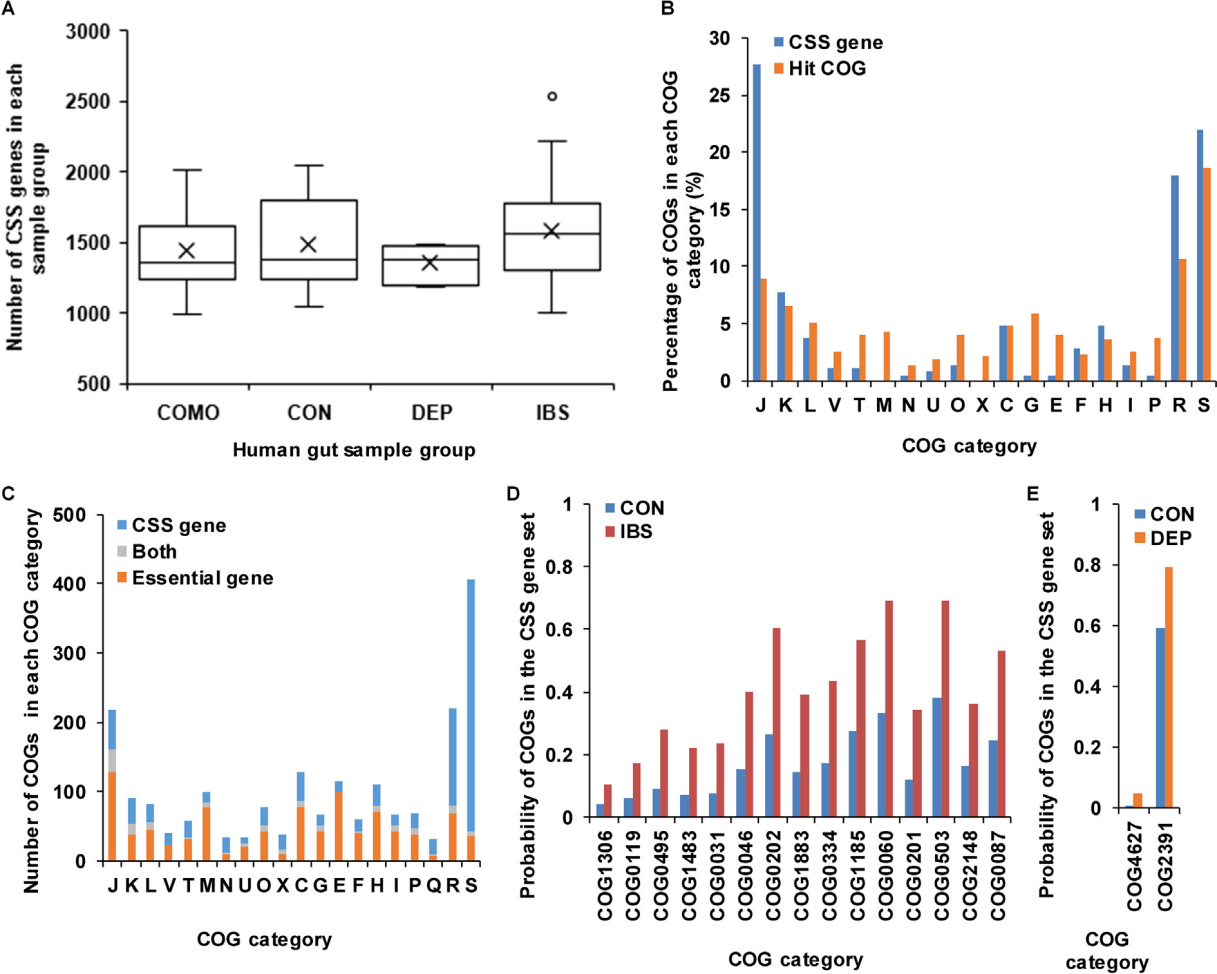
**Comparison of CSS genes in different human gut microbiota sample groups** **A.** Comparison of the numbers of CSS genes in COMO, CON, DEP, and IBS groups in human gut microbiota samples. The circles represent the outlier values and black crosses represent the average numbers of CSS genes in corresponding groups. **B.** Comparison of the distribution of CSS genes to all hit COGs in human gut microbiota samples. **C.** Comparison of the distribution of CSS genes and essential genes in all COG categories in human gut microbiota samples. COGs that are present in both the CSS gene set and essential gene set are shown in gray. Blue and orange bars indicate genes that only exist in the CSS gene set and DEG, respectively. **D.** Comparison of the COGs with significantly different probabilities to be CSS genes in CON and IBS groups. **E.** Comparison of COGs with significantly different probability to be CSS genes in CON and DEP groups. COMO, comorbidity; CON, health control; DEP, depression; IBS, irritable bowel syndrome; DEG, database of essential genes.

We also applied the workflow to identify CSS genes in human gut microbiota samples (Figure 8, Table S5). The numbers of CSS genes in comorbidity, health control, depression, and IBS groups were 1437.38 ± 292.20, 1483.35 ± 340.82, 1351.80 ± 133.90 and 1585.24 ± 371.92, respectively (Figure 8A). The numbers of CSS genes in human gut microbiota samples were obviously higher than those of AMD samples. This might be caused by the intrinsic complexity in the human gut microbial communities. We found that there were 226 COGs with high probabilities (probability = 1) to be CSS genes in all 54 samples. Compared to all hit COGs (Figure 8B), these 226 COGs were enriched in the categories J (translation, ribosomal structure and biogenesis), R (general function prediction only), and S (function unknown).

The differences between CSS genes and essential genes in human gut microbiota were much larger than those in the AMD samples (Figure 8C). In 3689 COGs with unique profile, there were 1125 essential genes and 1114 CSS genes (probability ≥ 0.5). However, only 153 COGs were shared by CSS genes and essential genes. The permutation test showed that the difference between CSS genes and essential genes was significant (permutation time = 10,000, *P* < 1E−4). In the 153 overlapped COGs, 20.92% (32/153) belonged to the category J (translation, ribosomal structure and biogenesis). 961 COGs, which were CSS genes but not found in DEG, were enriched in the categories S (function unknown), R (general function prediction only), J (translation, ribosomal structure and biogenesis), and C (energy production and conversion). 972 COGs, which were found in DEG but not in the CSS gene set, were mostly related to the categories J (translation, ribosomal structure and biogenesis), E (amino acid transport and metabolism), M (cell wall/membrane/envelope biogenesis), and C (energy production and conversion).

In addition, we also examined the COGs with significantly different probabilities to be CSS genes in four groups, including health control group, depression group, IBS group, and comorbidity group. Compared to samples in health control group, we found that 15 COGs were more likely to be CSS genes in IBS group (Student’s *t* test, *P* < 0.01) (Figure 8D, Table S6). Herein, COG2148 (sugar transferase involved in lipopolysaccharide biosynthesis) is involved in lipopolysaccharide biosynthesis; COG1883 (Na^+^-transporting methylmalonyl-CoA/oxaloacetate decarboxylase, beta subunit) is involved in the formation of oxaloacetate from pyruvate; and COG1483 (predicted ATPase, AAA+ superfamily) is a predicted ATPase. These three COGs were associated with saccharides, which might be utilized by intestinal microorganisms to produce gas, thus resulting in abdominal distension. COG0334 (glutamate dehydrogenase/leucine dehydrogenase), COG0119 (isopropylmalate/homocitrate/citramalate synthases), COG0495 (leucyl-tRNA synthetase), COG0031 (cysteine synthase), COG1185 (polyribonucleotide nucleotidyltransferase; polynucleotide phosphorylase), COG0060 (isoleucyl-tRNA synthetase), COG0503 (adenine/guanine phosphoribosyltransferase or related PRPP-binding protein), COG0046 (phosphoribosylformylglycinamidine (FGAM) synthase, synthetase domain), and COG0087 (ribosomal protein L3) were associated with the synthesis and metabolism of amino acids, nucleotides, and proteins. Amino acids are reported to be component of mucin in the intestinal epithelial barrier and thus associated with gut barrier function [42]. Herein, glutamine is an energy source of enterocytes [43]. Nucleotides are crucial for enterocytes in the development, maturation, and repair of intestine [43]. In addition, compared to samples in health control group, we found that 2 COGs were more likely to be CSS genes in depression group (Student’s *t* test, *P* < 0.01) (Figure 8E). Herein, COG4627 is a predicted *S*-adenosyl-l-methionine (SAM)-dependent methyltransferase, which transfers the methyl group from SAM to other substrates. For instance, catechol-*O*-methyltransferase, which belongs to SAM-dependent methyltransferase fold family [44], could methylate catechol compounds and inactivate the catechol neurotransmitter dopamine in the prefrontal cortex [45], thus it has many a times been suggested to be involved in affective disorders, such as depression [46].

## Discussion

To explore the mechanisms of microbial community adapting to the natural environments, we have proposed a novel replicator dynamics model, FCP model, based on functional genes of members within the community. With the attempt to integrate metagenomic sequences and environmental factors to quantify the motive power, we aim to circumvent the limitation of traditional dynamic models. Mainstream analyses in microbial ecology mostly build models with phenotypic parameters, which are often on the macroscopic scale. Herein, our model based on the molecular information and phenotypic parameters is built on both microscopic scale and macroscopic scale. Our study thus provides the insight into linking functional genes with the assembly of microbial communities. Using FCP model, the prediction matches the observed microbial community assemblage in both a relatively simple biological system and a complex one. The mean and variance of predicted values using our model are superior to those obtained using the MAP model, which has been proved to offer good prediction accuracy and widely used [21], [36]. The similarities of relative influence of environmental factors on population compositions obtained using different methods (*i.e.*, FCP, MAP and GBM models) also demonstrate the accuracy of our model.

Furthermore, we have proposed the concept of CSS genes, and developed an approach to select CSS genes in microbial community. We rebuild community at the functional level, not at the taxonomic level, which leads to good prediction performance. This suggests that the community structure is determined by functional genes rather than species, which might be helpful in holding the key to answer the fundamental question about what determines the composition of local communities [47], [48]. In addition, our data show that despite of the low relative abundances of archaea, they contribute greatly to maintaining the community structure. It is the minority, not the majority, that plays a far more important role in shaping community structure.

We take the metagenomic data of AMD microbiota as a typical microbial community to build the FCP model and identify CSS genes. An important focus of this study is to analyze how the CSS genes change during biofilm maturation. As the biofilm matures, the size of CSS gene set is decreased, partially due to the increased genomic diversity and physiological shifts. The clustering analysis illustrates that CSS genes in the different biofilm growth stages are distinct. Thus, an outline of CSS gene set could be sketched based on the developmental stage. CSS genes involved in genes about environmental stresses such as defense mechanisms have a higher probability of presence in the early succession stage, while genes encoding cell motility and membrane biogenesis are significantly increased at the late succession stage. Moreover, the enrichment of lipid transport and metabolism in mature biofilms with higher temperature is supported by studies about the changes of lipid composition in membranes of microorganisms under different temperature conditions [49]. In summary, we suggest that the top priority of AMD communities would be to resist pressures from extreme environments during early growth stage. With the development of AMD biofilms, the pressure resulting from competition for dwindling resources would be increased, thus cells try to move to places with more resources, leading to competition alleviation.

We also apply the model to human gut microbiota samples and identified CSS genes in each sample. We find that the numbers of CSS genes in human gut microbiota are much higher than those in AMD microbiota. Some COGs have significantly higher probability to be CSS genes in IBS group than health control group and they are enriched in the synthesis and metabolism of amino acids, nucleotides, proteins, and lipopolysaccharide. These substances are components of mucin in the intestinal epithelial barrier and thus important for gut integrity and gut barrier function repairment. In addition, these substances are gas producers, consistent with the abdominal distention in IBS group. We also find that a predicted SAM-dependent methyltransferase has significantly higher probability to be CSS gene in the depression group than in the health control group. This result is supported by many studies about the antidepressant properties of SAM [50], [51].

Although delineating the CSS gene set is still at a developing stage, our study about identifying CSS genes might help us to understand critical cellular processes that sustain communities. Also, it may be useful for designing addable gene circuitries to make an artificial self-sustainable community and treating diseases related to microbiota dysbiosis. There are also some limitations of our FCP model as following. (1) Too high dimensional data (for example, too many environmental factors or taxa) will pose a big challenge for prediction. (2) Metagenomics sequences are needed for the FCP model, and this costs much more than the models based on 16S rRNA sequences. (3) The prediction is limited if the biological system is largely influenced by the variables that we do not consider, such as undetected environmental factors. In this paper, we annotate genes with the COG database as an example in FCP model and CSS genes. In fact, we can use gene annotation from any other databases, such as the KEGG (Kyoto Encyclopedia of Genes and Genomes) database. In this study, we have applied our model and CSS gene selection method to AMD samples and human gut microbiota, and it could be expanded to other biological systems, such as soil systems and deep-sea systems.

## Material and methods

### Genomic data, gene prediction, and taxonomic classification

All genome sequences of the nine microorganisms from AMD samples were downloaded from the NCBI BioProject database (https://www.ncbi.nlm.nih.gov/bioproject/). The BioProject Accession Nos are listed as follows: PRJNA18795 (*Leptospirillum* group II), PRJNA37907 (*Leptospirillum* group III); PRJNA40089 (G-plasma); PRJNA29599 (A-plasma); PRJNA40091 (E-plasma); PRJNA40093 (I-plasma); PRJNA29595 (*Ferroplasma* type I); PRJNA29597 (*Ferroplasma* type II); and PRJNA38565 (ARMAN2). All metagenomic sequences of human gut microbiota and clinical parameters were generated by our lab or our collaborators [38]. We obtained 28 AMD samples and 60 human gut microbiota samples. Afterward, we removed samples with over half not determined environmental factors. Finally, we got 17 AMD samples and 54 human gut microbiota samples. The relative abundance of nine species accounts for 97.65 ± 0.79% of the total population of AMD samples after excluding unassigned sequences. In human gut microbiota samples, after excluding unassigned sequences, the relative abundance of taxa accounts for 98.26 ± 3.92%, 98.18 ± 2.05%, and 85.02 ± 12.10% of the total population of human gut microbiota samples at phylum, order, and genus level, respectively.

To carry out the analysis of the metagenomes, Quake [52] was used to detect and correct errors in the raw data. Prinseq [53] was used to filter out low quality reads. After that, InteMAP [54] was used to assemble these preprocessed reads into contigs. MetaGUN [55], a novel gene prediction tool, was used to predict protein coding genes, and MetaTISA [56] was then applied to revise translation initiation sites of predicted genes. PhymmBL [57], [58], the hybrid classifier combining analysis from both Phymm and BLAST, was used to perform taxonomic classification. Default parameters in these methods were used for the related analyses. Each predicted gene was annotated through searching COG database [33], [59], [60] by BLAST [61] with E-value = 1E−5.

### Statistical analyses of relationships

To quantify the influence of environmental factors on different microorganisms, we applied MRT and GBM analyses, which work well in interpreting the relationships between complicated ecological systems and their surroundings. To learn the relationships among the relative abundances of microorganisms, we used the CCREPE method. GBM, MRT, and CCREPE analyses were conducted with the gbm (with 5000 trees used for the boosting, 5-fold cross-validation and 3-way interactions), mvpart (with default parameters), and ccrepe (with default parameters) package in R statistical computing environment, respectively. GBM is a powerful machine learning method for regression and classification problems, and it can give a description of relative influence of several input variables on the target variations [35]. MRT analysis is a statistical technique that can be used to study complicated non-linear relationships by providing a taxonomy-supervised tree [34]. CCREPE takes the compositional effect into consideration and establishes corrections based on a null distribution. Cytoscape [62] was used for the biological network visualization.

### The FCP model

In the current study, we proposed a mathematical model based on the functional gene usage distribution to simulate and predict microbial population structure. This model was built on the modified replicator dynamics with variable population size. We described the interspecific interactions using the functional gene distribution. Then we used the interspecific interactions, combined with environmental factors, to quantify the fitness. In detail, for a community with *num* different kinds of species, we determined fitness with interspecific interactions and environmental filtering as follows:

$$f_{\mathrm{num}\times 1}=A_{\mathrm{num}\times num}x_{\mathrm{num}\times 1}+h_{\mathrm{num}\times 1}$$

After aligning genomes to all predicted peptides in COG database, we obtained the functional similarity through calculating the Pearson correlation coefficient between the distributions of functional genes in different species. This functional similarity matrix is denoted as **S**. The matrix of functional dissimilarity, A, is used to measure the benefit from functional cooperation between two microorganisms, we define A=L-S, where **L** is a matrix whose elements are 1. The matrix A shows that when microorganisms with similar functions meet, there would be likely to have relatively low benefit due to interspecific competition. h denotes the relationships between environmental factors and microbes, thereby presenting environmental filter tendency. Environmental data are stored in vector e. Column vector h is the product of matrix B and vector e, that is h=Be. Lasso regression, a regression analysis method capable of variable selection, was used to solve linear relationships between h and environmental factor e. Thus, we determined the critical motive for constructing a community by the functional gene distribution and environmental factors.

For a community with *num* different kinds of species, let *n_i_*, $i\in s=\left\{1,2,\cdots ,num\right\}$, be the number of the ith species at a given time. Then the population size is $N=\sum_{i\in s}n_{i}$ and the relative abundance of the ith species is $x_{i}=\frac{n_{i}}{N}$. The models are given by the following equation:

$$\left\{\begin{array}{c} {\dot{x}}_{i}=N^{c-1}\left(f_{i}x_{i}^{c}-x_{i}\sum_{j\in s}f_{j}x_{j}^{c}\right),i\in s\\ \dot{N}=N^{c}\sum_{j\in s}f_{j}x_{j}^{c}-dN \end{array}\right)$$

Here, ${\dot{x}}_{i}$ is the first derivative of $x_{i}$ versus time and $\dot{N}$ is that of N versus time. $f_{i}$ is the fitness of the ith species, and d is the death rate. Growth index c describes how much faster (c>1) or slower (0<c<1) the population size changes with time than exponential growth. Given what we considered is a microbial community under limited conditions, namely the growth of species is sub-exponential, we set 0<c<1. We chose this setting because microbial cells under extreme environments are reported to catabolize 10^4^- to 10^6^-fold slower than organisms in nutrient-rich cultures [63].

### Prediction using the FCP model

The FCP model was solved in MATLAB with the find minimum of constrained nonlinear multivariable function (FMINCON). The initial values were chosen based on the observed abundance distributions in AMD biofilms [32]. The initial values of human gut microbiota data were from the health control group. In fact, the FCP model is insensitive to the initial values of parameters. When other variables were kept the same and the initial relative abundances were altered on a large scale, 96.57% (482,829/500,000) of results were converged to a same one. The effect of initial values of population size N and death rate d on results is also limited. When we changed the initial values ofNfrom 1 to 1000,000, only numbers after the 4th decimal place of predictive results were influenced; and for d from 0.001 to 1, it was the 3th decimal place. Growth index c has some influence on community structure but little on the average results. Each sample we set 100 different and random initial values of h andc. The consistency of predictions of these 100 tries (the variance of Bray–Curtis similarity is 4.0±3.7%) show the robustness of our FCP model.

### CSS genes selection method

Due to the universality of functional redundancies, only several of genes play an important role in maintaining the stability of the microbial community structure. These genes are defined as CSS genes. Loss of CSS genes leads to significant changes of the community structure. Thus, we can pick up the CSS genes by testing the impact of genes on the community structure.

The FCP model allows us to quantify the contribution of each gene to the community structure. Perturbation calculations were used for measuring changes of the community structure. Bray–Curtis similarities between the perturbed community structures (small stochastic disturbances, 10,000 times in each sample) and unperturbed ones were calculated. If the Bray–Curtis similarity is beyond the threshold obtained by Student’s *t* test, we consider that there is a significant change in the microbial community structure after perturbation. Through screening genes one by one, we dropped genes which did not influence community structure significantly. To reduce the impact of the parameter selection in FCP model, we used 50 groups of parameters with good prediction and took the average of these 50 groups as the prediction output. At last, after repeating the steps across all samples, we figured out all CSS genes in the natural environments.

## Authors’ contributions

HZ and QW put forward the research plan and guided the project. XJ developed the mathematical modeling and analyzed data. XJ, XL, and LY assembled all the figures and tables and wrote the manuscript. CL wrote the program for CSS gene selection and performed the analysis. WC applied the FCP model to human gut microbiota data. All authors read the manuscript and approved the final edition.

## Competing interests

The authors have declared that no competing interests exist.

## References

[1] Larsen P., Hamada Y., Gilbert J. (2012). Modeling microbial communities: current, developing, and future technologies for predicting microbial community interaction. J Biotechnol.

[2] Gilbert J.A., Dupont C.L. (2011). Microbial metagenomics: beyond the genome. Annu Rev Mar Sci.

[3] Fuhrman J.A. (2009). Microbial community structure and its functional implications. Nature.

[4] Burke C., Steinberg P., Rusch D., Kjelleberg S., Thomas T. (2011). Bacterial community assembly based on functional genes rather than species. Proc Natl Acad Sci U S A.

[5] Almeida W.I., Vieira R.P., Cardoso A.M., Silveira C.B., Costa R.G., Gonzalez A.M. (2009). Archaeal and bacterial communities of heavy metal contaminated acidic waters from zinc mine residues in Sepetiba Bay. Extremophiles.

[6] Stojanović M.R., Biagi E., Heilig H.G.H.J., Kajander K., Kekkonen R.A., Tims S. (2011). Global and deep molecular analysis of microbiota signatures in fecal samples from patients with irritable bowel syndrome. Gastroenterology.

[7] Ng S.C., Lam E.F.C., Lam T.T.Y., Chan Y., Law W., Tse P.C.H. (2013). Effect of probiotic bacteria on the intestinal microbiota in irritable bowel syndrome. J Gastroenterol Hepatol.

[8] Jeffery I.B., O'Toole P.W., Öhman L., Claesson M.J., Deane J., Quigley E.M.M. (2012). An irritable bowel syndrome subtype defined by species-specific alterations in faecal microbiota. Gut.

[9] Naseribafrouei A., Hestad K., Avershina E., Sekelja M., Linløkken A., Wilson R. (2014). Correlation between the human fecal microbiota and depression. Neurogastroenterol Motil.

[10] Jeffreys B., Johnr T., Danielp R., Ingaa Z., Judee M. (2010). Factors affecting soil microbial community structure in tomato cropping systems. Soil Biol Biochem.

[11] Wei L., Shutao W., Jin Z., Tong X. (2014). Biochar influences the microbial community structure during tomato stalk composting with chicken manure. Bioresour Technol.

[12] Aciego Pietri J.C., Brookes P.C. (2009). Substrate inputs and pH as factors controlling microbial biomass, activity and community structure in an arable soil. Soil Biol Biochem.

[13] Maspolim Y., Zhou Y., Guo C., Xiao K., Ng W.J. (2015). The effect of pH on solubilization of organic matter and microbial community structures in sludge fermentation. Bioresour Technol.

[14] Wellborn G.A., Skelly D.K., Werner E.E. (1996). Mechanisms creating community structure across a freshwater habitat gradient. Annu Rev Ecol Syst.

[15] Luo H., Lin Y., Gao F., Zhang C., Zhang R. (2013). DEG 10, an update of the database of essential genes that includes both protein-coding genes and noncoding genomic elements. Nucleic Acids Res.

[16] De Y., Dong C., Cao Y., Wang X., Yang X. (2017). Genome-wide sequence transposon insertion sites and analyze the essential genes of *Brucella melitensis*. Microb Pathog.

[17] Goodman A.L., McNulty N.P., Zhao Y., Leip D., Mitra R.D., Lozupone C.A. (2009). Identifying genetic determinants needed to establish a human gut symbiont in its habitat. Cell Host Microbe.

[18] Zhang Y.J., Ioerger T.R., Huttenhower C., Long J.E., Sassetti C.M., Sacchettini J.C. (2012). Global assessment of genomic regions required for growth in *Mycobacterium tuberculosis*. PLoS Pathog.

[19] Minkenberg B., Xie K., Yang Y. (2017). Discovery of rice essential genes by characterizing a CRISPR-edited mutation of closely related rice MAP kinase genes. Plant J.

[20] Larsen P.E., Gibbons S.M., Gilbert J.A. (2012). Modeling microbial community structure and functional diversity across time and space. FEMS Microbiol Lett.

[21] Larsen P.E., Field D., Gilbert J.A. (2012). Predicting bacterial community assemblages using an artificial neural network approach. Nat Methods.

[22] Stein R.R., Bucci V., Toussaint N.C., Buffie C.G., Ratsch G., Pamer E.G. (2013). Ecological modeling from time-series inference: insight into dynamics and stability of intestinal microbiota. PLoS Comput Biol.

[23] Marino S., Baxter N.T., Huffnagle G.B., Petrosino J.F., Schloss P.D. (2014). Mathematical modeling of primary succession of murine intestinal microbiota. Proc Natl Acad Sci U S A.

[24] Trosvik P., Rudi K., Naes T., Kohler A., Chan K.S., Jakobsen K.S. (2008). Characterizing mixed microbial population dynamics using time-series analysis. ISME J.

[25] Trosvik P., Stenseth N.C., Rudi K. (2010). Convergent temporal dynamics of the human infant gut microbiota. ISME J.

[26] Gerber G.K. (2014). The dynamic microbiome. FEBS Lett.

[27] Schuster P., Sigmund K. (1983). Replicator dynamics. J Theor Biol.

[28] Li X., Pietschke C., Fraune S., Altrock P.M., Bosch T.C.G., Traulsen A. (2015). Which games are growing bacterial populations playing?. J R Soc Interface.

[29] Eyre-Walker A., Keightley P.D. (2007). The distribution of fitness effects of new mutations. Nat Rev Genet.

[30] Laughlin D.C., Joshi C., van Bodegom P.M., Bastow Z.A., Fulé P.Z. (2012). A predictive model of community assembly that incorporates intraspecific trait variation. Ecol Lett.

[31] Guo J., Wang Q., Wang X., Wang F., Yao J., Zhu H. (2015). Horizontal gene transfer in an acid mine drainage microbial community. BMC Genomics.

[32] Mueller R.S., Denef V.J., Kalnejais L.H., Suttle K.B., Thomas B.C., Wilmes P. (2010). Ecological distribution and population physiology defined by proteomics in a natural microbial community. Mol Syst Biol.

[33] Galperin M.Y., Makarova K.S., Wolf Y.I., Koonin E.V. (2014). Expanded microbial genome coverage and improved protein family annotation in the COG database. Nucleic Acids Res.

[34] De'ath G. (2002). Multivariate regression trees: a new technique for modeling species-environment relationship. Ecology.

[35] Natekin A., Knoll A. (2013). Gradient boosting machines, a tutorial. Front Neurorobot.

[36] Kuang J., Huang L., Chen L., Hua Z., Li S., Hu M. (2013). Contemporary environmental variation determines microbial diversity patterns in acid mine drainage. ISME J.

[37] Spiller R., Aziz Q., Creed F., Houghton L., Hungin P., Jones R. (2007). Guidelines on the irritable bowel syndrome: mechanisms and practical management. Gut.

[38] Liu Y., Zhang L., Wang X., Wang Z., Zhang J., Jiang R. (2016). Similar fecal microbiota signatures in patients with diarrhea-predominant irritable bowel syndrome and patients with depression. Clin Gastroenterol Hepatol.

[39] Cryan J.F., Dinan T.G. (2015). More than a gut feeling: the microbiota regulates neurodevelopment and behavior. Neuropsychopharmacology.

[40] Mendiola M.V., Bernales I., De L. (1994). Differential roles of the transposon termini in IS91 transposition. Proc Natl Acad Sci U S A.

[41] Benjak A., Forneck A., Casacuberta J.M. (2008). Genome-wide analysis of the “cut-and-paste” transposons of grapevine. PLoS One.

[42] Jalanka-Tuovinen J., Salojärvi J., Salonen A., Immonen O., Garsed K., Kelly F.M. (2014). Faecal microbiota composition and host-microbe cross-talk following gastroenteritis and in postinfectious irritable bowel syndrome. Gut.

[43] McCauley R., Kong S.E., Hall J. (1998). Glutamine and nucleotide metabolism within enterocytes. JPEN J Parenter Enteral Nutr.

[44] Ma Z., Liu H., Wu B. (2014). Structure-based drug design of catechol-*O*-methyltransferase inhibitors for CNS disorders. Br J Clin Pharmacol.

[45] Tunbridge E.M., Bannerman D.M., Sharp T., Harrison P.J. (2004). Catechol-*O*-methyltransferase inhibition improves set-shifting performance and elevates stimulated dopamine release in the rat prefrontal cortex. J Neurosci.

[46] Aberg E., Fandiño-Losada A., Sjöholm L.K., Al E. (2011). The functional Val158Met polymorphism in catechol-*O*-methyltransferase (COMT) is associated with depression and motivation in men from a Swedish population-based study. J Affect Disord.

[47] Webb C.T., Hoeting J.A., Ames G.M., Pyne M.I., Poff N.L.R. (2010). A structured and dynamic framework to advance traits-based theory and prediction in ecology. Ecol Lett.

[48] Laliberté E., Shipley B., Norton D.A., Scott D. (2012). Which plant traits determine abundance under long-term shifts in soil resource availability and grazing intensity?. J Ecol.

[49] Allen E.E., Bartlett D.H. (2002). Structure and regulation of the omega-3 polyunsaturated fatty acid synthase genes from the deep-sea bacterium *Photobacterium profundum* strain SS9. Microbiology.

[50] Mischoulon D.F.M. (2002). Role of S-adenosyl-l-methionine in the treatment of depression: a review of the evidence. Am J Clin Nutr.

[51] Bressa G.M. (1994). *S*-adenosyl-L-methionine (SAMe) as antidepressant: meta-analysis of clinical studies. Acta Neurol Scand.

[52] Kelley D.R., Schatz M.C., Salzberg S.L. (2010). Quake: quality-aware detection and correction of sequencing errors. Genome Biol.

[53] Schmieder R., Edwards R. (2011). Quality control and preprocessing of metagenomic datasets. Bioinformatics.

[54] Lai B., Wang F., Wang X., Duan L., Zhu H. (2015). InteMAP: Integrated metagenomic assembly pipeline for NGS short reads. BMC Bioinformatics.

[55] Liu Y., Guo J., Hu G., Zhu H. (2013). Gene prediction in metagenomic fragments based on the SVM algorithm. BMC Bioinformatics.

[56] Hu G., Guo J., Liu Y., Zhu H. (2009). MetaTISA: metagenomic translation initiation site annotator for improving gene start prediction. Bioinformatics.

[57] Brady A., Salzberg S.L. (2009). Phymm and PhymmBL: metagenomic phylogenetic classification with interpolated Markov models. Nat Methods.

[58] Brady A., Salzberg S. (2011). PhymmBL expanded: confidence scores, custom databases, parallelization and more. Nat Methods.

[59] Tatusov R.L., Fedorova N.D., Jackson J.D., Jacobs A.R., Kiryutin B., Koonin E.V. (2003). The COG database: an updated version includes eukaryotes. BMC Bioinformatics.

[60] Tatusov R., Galperin M., Natale D., Koonin E.V. (2000). The COG database: a tool for genome-scale analysis of protein functions and evolution. Nucleic Acids Res.

[61] Altschul S., Gish W., Miller W., Myers E.W., Lipman D.J. (1990). Basic local alignment search tool. J Mol Biol.

[62] Shannon P., Markiel A., Ozier O., Baliga N.S., Wang J.T., Ramage D. (2003). Cytoscape: a software environment for integrated models of biomolecular interaction networks. Genome Res.

[63] Hoehler T.M., Jørgensen B.B. (2013). Microbial life under extreme energy limitation. Nat Rev Microbiol.

